# Promises and pitfalls of digital credit: Empirical evidence from Kenya

**DOI:** 10.1371/journal.pone.0255215

**Published:** 2021-07-23

**Authors:** Constantin Johnen, Martin Parlasca, Oliver Mußhoff

**Affiliations:** Department of Agricultural Economics and Rural Development, University of Goettingen, Goettingen, Germany; Szechenyi Istvan University: Szechenyi Istvan Egyetem, HUNGARY

## Abstract

Digital credit is a recent innovation that raises hopes of improving credit access in developing countries. However, up until now, empirical research on the extent to which digital credit actually reaches people who are otherwise excluded from conventional credit markets and whether increased credit access is sustainable or threatened by high default and blacklisting rates is very scarce. Using representative data from Kenya, this article shows that digital credit increases borrowing opportunities, including for people less likely to otherwise have credit access in the conventional credit markets. However, we find that digital credit borrowing is also responsible for 90% of all blacklistings, which is partially driven by higher default rates in the digital credit market but also by a higher probability that digital credit defaults lead to blacklisting of the borrower, compared to defaults in other credit markets.

## 1. Introduction

In a rising number of low-and middle-income countries, digital credit is an increasingly prominent source of borrowing [1, 2]. Digital lenders can leverage non-traditional data (e.g. mobile phone usage data) instead of financial histories for credit assessment and deliver consumption credit via widely-spread mobile money networks [3]. Thereby digital credit becomes particularly valuable for the considerable share of individuals in developing countries who live remotely or lack financial footprints. Congruently, three years after the launch of the first digital credit product in Kenya in 2012, this from of credit was already accessible in 16 countries [1]. The rapid diffusion of digital credit has led to the general notion that this form of credit is suitable to overcome credit-constraints of marginalized groups. It was further assumed that digital credit could constitute a pathway to conventional credit, through the creation of financial footprints for the formerly excluded [3].

Despite the obvious potential of the easy-to-access consumption credit, it is far from being agreed on that digital credit invariably enhances welfare. The ease of access itself, in combination with potentially financially inexperienced borrowers, might induce credit take-up that leads to overborrowing, default and vicious circles of debt [4–6]. Indeed, in a case study in Mexico, digital credit displayed default rates of almost 27% [7]. Similarly, by 2017 in Kenya alone, an estimated 2.7 million digital borrowers have been reported to one of the credit reference bureaus (CRBs) for defaulting [8]; a red flag that often results in a long-term exclusion from all (semi-) formal lenders and is consequently termed “blacklisting” [5, 8].

The stylized portrayal of digital credit alludes that this form of credit represents both an opportunity and a risk. This is also reflected by heated public discussions and a variety of far-reaching policy measures undertaken in that sector in recent years, including a temporary suspension for digital lenders to be entitled to report defaulters in Kenya [9]. However, the scientific foundation for such decisions remains scant, as previous literature about digital credit has mainly focused on case studies [7, 10–12] or remains theoretical, qualitative, or anecdotal. To fill this gap, the present study investigates the following questions: Does digital credit improve credit access for people who are less likely to access conventional formal credit and thereby create a pathway to conventional formal credit? How do default rates and the subsequent reporting of default differ between digital and conventional credit?

To address these questions, this article uses data from three nationally representative cross-sectional surveys from Kenya. Kenya is a particularly suitable country with its´ background for this article, given that the country has the highest distribution of digital credit among all developing and emerging economies. The insights are, however, also relevant for other low- and middle-income countries, where digital credit has recently become available to consumers as well. Based on descriptive tools, the results suggest that in Kenya, digital credit has indeed increased borrowing opportunities substantially, also for individuals, who are expected to have lesser access to conventional formal credit, but not constituted a pathway to conventional formal credit. We also find that blacklisting rates are considerably higher in the digital compared to the (semi-) formal credit markets, driven by both high default rates and stricter reporting of defaults in the digital credit market compared with other credit markets. These results offer important insights into the broader effects of digital credit in Kenya and emphasize that changes in the regulation and in design of digital credit are needed so that digital credit can indeed provide a sustainable source of credit.

The remainder of this study is organized as follows. Section 2 provides a literature review. Section 3 describes the data. In Section 4, we analyze how many and which people in Kenya use digital credit. In Section 5, we investigate two critical pitfalls of digital credit, namely default and blacklisting. In Section 6, we derive policy implications and conclude.

## 2. Literature review

### 2.1 Background of digital credit

Digital credit is built on the infrastructure of mobile money, a mobile financial service (MFS) that led to a significant increase in income and reduction in poverty rates by enabling mobile phone users to transfer money via the ubiquitous mobile networks [13, 14]. Worldwide more than 1.2 Billion mobile money users exist, showing both the existence of high demand and a suitable infrastructure to use MFS in many developing countries [2].

The latter is exploited by digital credit lenders worldwide insofar that application for and delivery of digital credit occurs entirely via mobile phones/devices. Accordingly, three key features that define digital credit have been derived [3, 15, 16]. First, digital credit is instant; this means that the time span between application and approval is typically less than 24 hours. Second, digital credit is automated; this refers to the automated credit approval process, which is undertaken by algorithms, based on non-traditional data (such as mobile money or phone usage data). Third, digital credit is remote; this refers to the possibility of accessing digital credit from wherever the borrower has access to mobile network [16]. Digital credit hence encompasses multiple dimensions, distinguishing itself from other related digital lending models, such as digital peer-to-peer lending, in which peers (not algorithms) assess credit worthiness [17].

Next to the attempt of a universal definition of digital credit, the status quo of digital credit can be described as short term, low-sized consumption credit, with high interest rates [15]. The three key features, as well as the status quo characteristics make digital credit especially useful to smooth consumption (for instance after shock), when credit is needed immediately to cover basic expenses; whereas the short repayment periods and small-loan sizes render digital credit in its typical form unsuitable for long-term investments. Congruently, Bharadwaj et al. (2019) find that digital credit significantly increased resilience after shocks, albeit transformative effects were not found [10]. Similarly, a study conducted in China finds that online consumer credit significantly increases consumption in poorer and less developed areas [18]. These findings are also in line with studies on small-sized consumption credit in the conventional market [19].

At the same time consumer credit may be used for unproductive expenditures [4, 19]. Indeed, recent studies in this field show a positive relationship between digital credit usage and mobile betting [12]. The usage of digital credit for nonproductive purposes in combination with behavioral biases may lead to overborrowing among consumers, creating vicious circles of debt repayments [4, 6, 19, 20]. The risk of default may be further exacerbated by deliberate default, caused by a lack of typical contract enforcement mechanisms for digital lenders, such as the seizure of collateral [21]. Accordingly, case studies and grey literature report high default rates among digital credit users in a variety of countries, including Kenya, Mexico and Tanzania [7, 22].

### 2.2 Credit markets in Kenya

In many low-and middle-income countries, including Kenya, credit markets are largely fragmented and typically defined as formal, semi-formal and informal. The informal credit market, in contrast to the formal, is not regulated by a central monetary authority, whereas the semi-formal market encompasses both formal and informal features [23, 24]. These fragmented credit markets have emerged due to large obstacles for many individuals in being able to access formal credit [25] and have consequently evolved to serve different groups of individuals with distinct characteristics [23]. In the present study, we define conventional credit from regulated banks and the government as formal credit. While the formal financial sector in Kenya is considered among the most developed in sub-Saharan Africa, in 2010 the vast majority (78%) of Kenyans were not served by conventional formal financial institutions [24].

In an attempt to compensate for the failures of the formal credit market, i.e. to enable poorer, financially excluded individuals to borrow, Micro Financial Institutions and Savings and Credit Cooperative Organizations have emerged [24]. In addition to the two aforementioned financial institutions we also consider Chamas (a form of private group lending) as semi-formal, insofar they are officially registered. The semi-formal sector plays an important role in Kenya; by 2010 this sector has reduced the credit access gap for approximately 18% of Kenya´s adult population [24]. The effects of microfinance credit however remain contested due to high debt stress and little transformative effects; leading to a considerable amount of research investigating how to reduce debt stress in that sector [26, 27]. In addition, as semi-formal lenders also often require the provision of collateral or to undertake long journeys to qualify/apply for credit, a considerable share of the remote population is left credit constrained from both the formal and semi-formal credit market.

Digital credit largely overcomes these constraints. Unsurprisingly, digital credit take-up was rapid, surpassing the volume of conventional credit in Kenya already in 2015 [28]. Over the past few years, digital credit in Kenya has diversified to encompass differing business models that span a variety of regulatory classifications, some which would fall under the definition of the formal, some under the semi-formal credit market. While this constitutes a development that implies far-reaching regulatory challenges [3], the definition of digital credit provided in Section 2.1 is applicable to all regulatory forms. Therefore, the merits of further splitting up digital credit market(s) in the present study do not seem to balance out the added complexity, i.e. the regulatory classifications of different digital credit lender´s business models are not considered in the present study. It should further be noted that the informal credit market (e.g. lending from friends, shylocks, shopkeepers etc.) is not included in the present study, as default rates are unknown and blacklisting is not possible.

### 2.3 The role of credit reference bureaus for consumer credit

**Fig 1:**
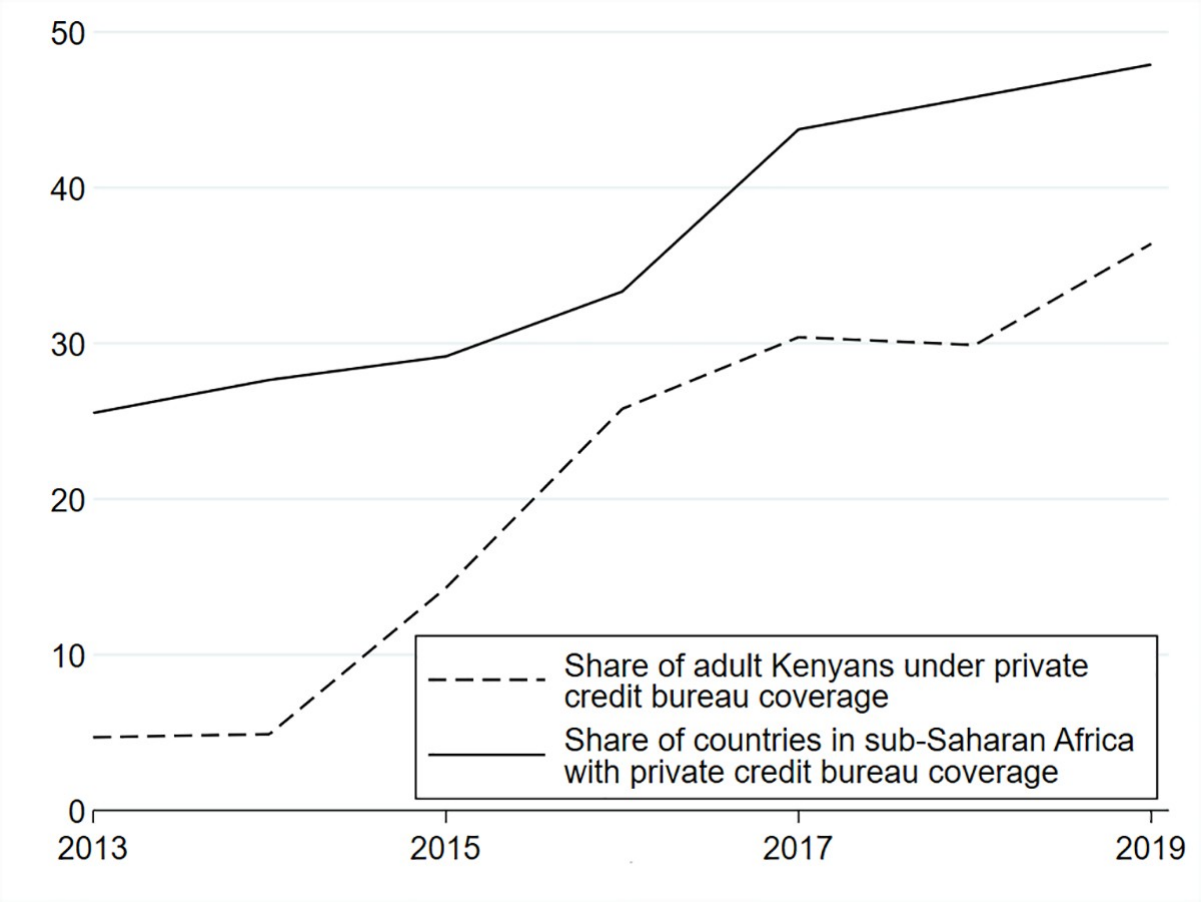
Coverage of private credit bureaus. Own calculations based on World Development Indicators from 2013–2019.

In principle, CRBs which make information on repayment histories available to lenders benefit private credit markets in developing economies [29–32]. CRBs have been related to an increase in money supply and financial activity [33]. Over the last years, private credit bureau coverage in sub-Saharan Africa has increased substantially both at the extensive and intensive margins. Fig 1 shows that the share of sub-Saharan countries with some private credit bureau coverage nearly doubled from 25% in 2013 to 48% in 2019. In the same time span, the share of Kenya’s population covered by private credit bureaus increased from less than 5% to 36%.

In consumer credit markets, which are characterized by short‐term interactions and limited legal protection of borrowers, such credit registries can be particularly important, due to their function as a contract enforcement mechanism. A negative report can be accessed by other (semi-) formal lenders, with potentially severe and extensive consequences for the defaulter [5]; even a single reported default can decrease access to future credit from any type of (semi-) formal lender, regardless of the overall borrowing history and therefore imply a long-term de facto exclusion from the (semi-) formal credit markets [5, 8, 34]. In order to be able to access credit again, blacklisted individuals typically need to pay all outstanding amounts of those credit, which are negatively listed, and, in addition, obtain a clearance certificate from a CRB. In Kenya, such clearance certificates usually entail a fixed fee of around USD20 [34]. As digital lenders lack other typical enforcement mechanisms, such as collateral, strict reporting to CRBs might therefore be used to compensate for this deficit.

## 3. Materials and methods

This article uses data based on the three most recent FinAccess household surveys from 2013, 2016, and 2019. The FinAccess household surveys are repeated cross-sectional surveys and represent a collaboration between the Kenya National Bureau of Statistics (KNBS), Financial Sector Deepening Trust (FSD) Kenya and the Central Bank of Kenya (CBK). Sampling contains three levels, to achieve a statistically valid, nationally representative sample of individuals aged 16 and above. The first level encompasses the selection of clusters; the second level the selection of households in each cluster; and in the third level an individual aged 16 or above is randomly selected within the household. Selection is done without replacement [35–37].

The surveys are administered through face-to-face interviews in English and translations in several local languages. The enumerators work in groups of 4 or 5 while in the field, with an experienced team leader responsible for each group. In addition, quality control is ensured through external and internal mechanisms.

The 2013 FinAccess household survey was conducted during the time period of October 2012 to February 2013 and resulted in 6,449 respondents. Given the target sample size of 8,520, the respondents represent a success rate of 76%. The 2016 FinAccess household survey was conducted during the time period of August to October 2015 and resulted in 8,865 respondents. Given the target sample size of 10,008, the respondents represent a success rate of 87%. The 2019 FinAccess household survey was conducted during the time period of October to December 2018 and resulted in 8,669 respondents. Given the target sample size of 11,000, the respondents represent a success rate of 79%. The three surveys are publicly available; links and references can be found in the data availability statement.

We excluded all individuals, who are aged below 18 years, since this is the age for legally being allowed to take-up credit. This results in final sample sizes of 6,186 individuals (2013), 8,208 individuals (2016), and 8,267 (2019). In addition, we conducted weighting adjustments for each survey in order to provide estimates that are representative of the target population at a national level, as sample allocation was not proportional to the size of the strata and some of the sampled households did not respond to the interviews, while others could not be accessed.

A pre-condition to be able to investigate effects of digital credit take-up, including default and backlisting, on a national level is that the diffusion of digital credit is not too low, since default and even more so blacklisting only represent a fraction of digital credit take-up. Kenya was therefore chosen as the study region, since digital credit diffusion in Kenya is particularly high. Nevertheless, the results are likely to be applicable in many other low-and middle-income countries, where this rather expensive form of low-sized consumption credit is increasingly popular among individuals with little financial experience, including India, Mexico, or the Ivory Coast [2, 7, 38].

## 4. Promises of digital credit

### 4.1 Dissemination of digital credit in Kenya over time

**Fig 2:**
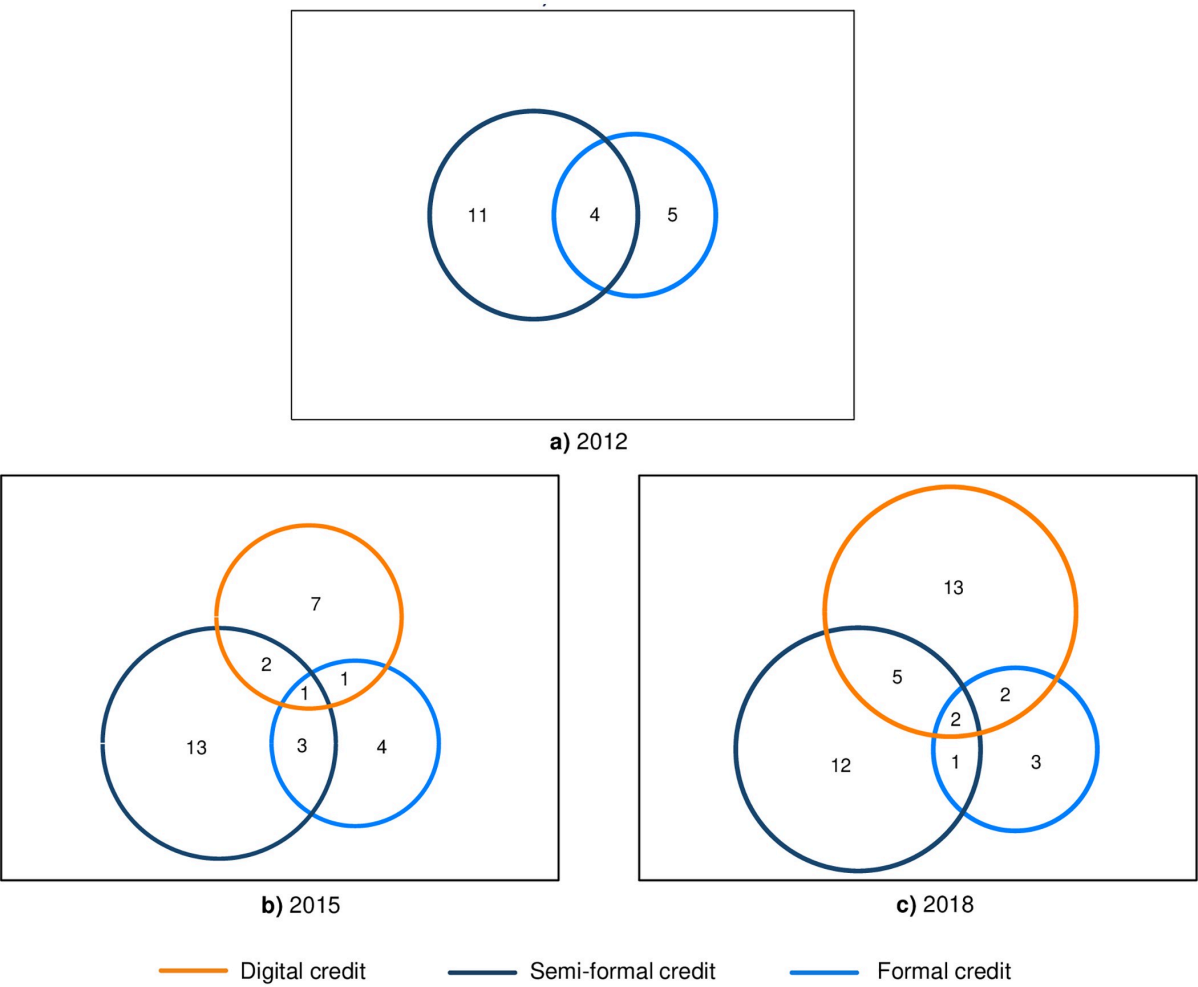
Development of the shares of adults obtaining credit from various sources. Within the Venn diagrams, proportions are shown that relate to the trimmed area. For instance, in a), 11% of adult Kenyans have borrowed solely from semi-formal lenders, 5% have borrowed solely from formal lenders and 4% have borrowed from both formal and semi-formal lenders. The rectangle framing each sub-figure represents the entire adult population in that year, i.e. 100%. Source: Own calculations based on the FinAccess Survey Data published in 2013, 2016, and 2019.

Based on the three FinAccess household surveys, we estimated the share of adult Kenyans, who have (or have had) credit access to at least one of the three credit markets, digital, semi-formal and formal. During a time span of six years (2012–2018), the share of adult Kenyans, who have (or have had) credit access to at least one of these markets nearly doubled from 19.8% to 38.7% (Fig 2A & 2C). While the share of people borrowing from the formal or semi-formal market remained relatively stable during that time period, the proportion of people taking a digital credit grew from zero percent in 2012 to more than 21% in 2018. In less than six years, the digital credit market has therefore already transformed into the largest credit market in terms of the number of borrowers, and—more importantly—served a considerable part of the adult population (12.8%) exclusively. Given that digital credit is typically not a substitute for other forms of credit [10], this suggests that the digital credit market has opened borrowing opportunities for a non-negligible share of Kenya’s population.

Conventional lenders typically require some form of financial history in order to decide on a borrower’s credit worthiness, leaving a considerable share of individuals excluded from these credit markets. Given that digital lenders can access and utilize indicators such as mobile phone usage -which is nearly ubiquitous in Kenya-, digital lenders are in theory able to expand credit access to a much broader spectrum of individuals regardless of their financial history status. This further implies that individuals might be able to start building a financial history through digital borrowing and consequently digital credit might constitute a pathway for people to help them gain access also to formal credit [3]. However, Fig 2 shows that while digital credit access grew considerably, the share of individuals who had access to the formal credit market remained rather constant over time. We view this as an indication that digital credit—at least so far—has not been a substantive pathway to formal credit.

An important requirement for such a pathway is that digital lenders indeed fully report all positive and negative borrowing behavior to a credit bureau. Yet, previous grey literature has argued that digital lenders’ reports may not be fully exhaustive, even though existing regulations require them to do so [5, 39]. When these regulations are not fully enforced, digital lenders face a trade-off between the benefit from information sharing as an enforcement mechanism and the costs of losses in extractable rents. For digital lenders, in contrast to conventional lenders, the threat of blacklisting may be relatively more valuable as an enforcement mechanism, as digital lenders have lesser access to other enforcement mechanisms [21]. However, at the same time, the losses in extractable rents may also be relatively larger for digital lenders compared to conventional lenders. This is because for digital lenders, borrower-lender relationships are particularly valuable, since the development of appropriate algorithms to process data for credit evaluation typically involve considerable investments. Publicly available data on a borrower´s credit behavior would allow “outside” (digital) lenders to free-ride on these investments [3]. In a state where regulations are not fully enforced it may hence be ideal from a digital lender’s perspective to strictly report defaulters (further discussed in section 5.2), while underreport timely repayments; the latter, in turn, is therefore a plausible hindrance for the pathway from digital credits to formal credits.

### 4.2 Who takes digital credit?

Digital credit is hoped to be a form of credit that is accessible also to those who are less likely to obtain formal credit. This hope is mainly based on the assumption that poorer people are more likely to leave digital footprints compared to financial footprints, where the former can be sufficient for digital lenders to assign credit worthiness. However, it remains unknown whether the digital footprint of poorer individuals are also large enough for algorithms to assign credit scores, as poorer individuals tend to use their mobile devices less often [11]. In addition, even if data are sufficient for an initial credit score in the digital credit market, algorithms might discriminate against vulnerable groups [40, 41].

In order to show which people in society use digital credit, Table 1 presents socio-economic characteristics of those, who have obtained a digital credit and compares them to users of formal and semi-formal credit. In particular, we show characteristics that are typically associated with lower credit access opportunities, such as having a low income and no or an irregular occupation, as well as other indicators of vulnerability [42–45]. Since most key characteristics about borrowing behavior in general and digital credit in particular are only available for the FinAccess Survey 2019, the remainder of the analysis focuses on this particular survey.

**Table 1:** Borrower characteristics in the formal-, semi-formal and digital credit market.

Borrower Characteristics	Formal	Semi-Formal	Digital
N^1^	359	1,129	1,224
**Socio-Demographics**			
Age [years]	39 (14)	41 (13)	35 (12)
Share of users in the female population [%]	3	17	16
Share of users in the male population [%]	8	13	21
Financial Literacy^2^ [%]	82	55	65
Monthly income [1.000 KES ~ 9,8 USD]	31 (35)	18 (24)	17 (22)
Urban [%]	60	45	64
**Highest Education Level Completed** [%]			
No education	7	21	10
Primary education only	14	35	31
Secondary education or more	78	44	58
**Main Occupation** [%]			
Casual	5	15	21
Employed	35	23	24
**Vulnerability** [%]			
Household has gone without food at least once in the last year [1 = yes]	11	21	19
Household has gone without medicine at least once in the last year [1 = yes]	14	27	25

Note:

^1^The markets are not mutually exclusive. N_total_ = 2,194

^2^ Financially literacy is a dummy that indicates whether the person correctly calculated the interest rate of a hypothetical credit. Standard deviations are shown in parentheses. Source: Own calculations based on FinAccess Survey 2019.

For several characteristics, substantial differences between users of digital credit and users of formal credit exist. As shown in Table 1, the average income of formal credit borrowers is almost twice the average income of digital credit borrowers and, similarly, the likelihood that an individual has gone without food or medicine in the past twelve months is about half for formal credit borrowers compared to digital borrowers. It is also noteworthy that digital credit borrowers are more likely to gain their main income through casual work (21%) compared to formal credit borrowers (5%) and less likely to be employed than formal credit borrowers (24% and 35%). These differences suggest that digital credit is, in fact, used by those who are less likely to obtain formal credit due to low and irregular income.

Between borrowers from the semi-formal credit market and borrowers from the digital credit market, differences in incomes, occupation and vulnerability are much less pronounced. However, it is noteworthy that borrowers in the semi-formal credit market are over-proportionally likely to be female. This is consistent with previous literature showing that semi-formal lenders seem to favor woman in rural areas [42]. In contrast, the digital credit market is accessed by 21 percent of the male population, while only 16 percent of the female population do so. It is also noteworthy that the digital credit market has the highest share of users living in urban areas among all three credit markets. Since access to digital credit does not require the individual to personally travel and interact with a bank, digital credit is often assumed to be particularly valuable for the rural population. This argument does not find support in the here-presented data.

The fact that digital credit is not only used by a large share of the Kenyan population, but also by people who are more likely to face difficulties in obtaining formal or semi-formal credit due to income, education or gender indicates that the introduction of digital credit nine years ago has in fact led to a strong increase in access to credit among Kenya’s population. However, the sustainability of this digital credit induced increase in credit access is questionable, since lower levels of income and higher probabilities of having irregular incomes that separate borrowers from the digital credit market and the formal credit market are associated with higher probabilities of credit default [23, 46]. The fact that more risky borrowers now have access to credit could result in higher rates of default, overborrowing, negative reporting to a credit bureau and eventual exclusion from the credit market due to blacklisting.

## 5. Pitfalls of digital credit

### 5.1 Default and blacklisting due to digital credit

The 2019 FinAccess survey entailed a unique set of questions regarding borrowing behavior, i.e. whether individuals have defaulted or been blacklisted within 12 months prior to the survey. Based on those respondents who have indicated to have used at least one credit within the 12 months prior to the survey and are aged above 18 years (n = 2,194), we estimated probabilities of defaulting on a certain loan and the probabilities of being blacklisted. Default does not necessarily have to lead to blacklisting, since blacklisting also requires an action of the lender, i.e. a report of the default to a CRB. As mentioned earlier there are theoretical reasons and empirical indications to assume that even regulated lenders, *de facto*, report borrowing behavior only fragmentary [5, 39].

The results in Table 2 show that default rates are considerably higher when borrowing digitally (13.8%) compared to both borrowing in the semi-formal (7.1%) or formal credit market (6.4%). The substantial default rate in the digital credit market in itself already raises questions about the long-term sustainability of digital credit in their current form both from the borrowers´ and the lenders´ perspective. Understanding this comparatively high default rate in that credit market is paramount for the design of efficient measures to reduce default rates.

**Table 2:** Default rate and share of reported defaults by credit type (percent).

Credit Type	Defaults	Reported Defaults	Blacklisting
Digital	13.8	38.4	5.3
	(1.2)	(4.4)	(0.8)
Formal	6.4	22.7	1.4
	(1.6)	(11.7)	(0.8)
Semi-Formal	7.1	2.1	0.1
	(0.9)	(1.6)	(0.1)

Source: Proportions are shown. Linearized standard errors are shown in parentheses. Own calculations based on FinAccess Survey 2019

In Table 1 we showed that digital credits indeed seem to be exercised, to a large extend, by individuals, who might otherwise be excluded from the formal credit market for being considered risky borrowers [23, 46, 47]. This very self-selection of risky borrowers in the digital credit market, in turn, might increase default rates of digital credit. However, borrowers, who receive low creditworthiness scores from formal lenders might also self-select into semi-formal markets. This argument is supported by the fact that characteristics, which are often associated with risky borrowers, such as low income or high vulnerability are largely similar to digital credit borrowers and semi-formal credit borrowers. Still, default rates in the digital credit market are roughly twice the default rates in the semi-formal market. This suggests that also reasons other than mere self-selection of risky borrowers are responsible for higher default rates in the digital credit market, including differing credit-usage patterns [3, 10], terms-and conditions of digital credit (including excessively high fees) [15], strategic default [21] and deliberate confusion of borrowers [3].

Furthermore, Table 2 also shows that probabilities of being blacklisted are considerably higher in the digital credit market (5.3%) compared to formal (1.4%) and semi-formal (0.1%) credit markets. Given that high blacklisting rates of digital credit can become severe obstacles to sustainable credit access for many borrowers, the next section provides insights into the second mechanism of blacklisting, i.e. the lender´s action to report the negative borrowing behavior.

### 5.2 Reporting of defaults

While it is not entirely clear yet to which extend high default rates in the digital credit market are borrower or lender driven, it is plausible to assume that the decision to report a default, lays in the sphere of the lender entirely; with different lenders potentially having differing incentives to actually report a default or use other measures instead [48]. As mentioned earlier, digital lenders face a trade-off between losses in extractable rents due to information sharing and information sharing as an enforcement mechanism. However, solving this tradeoff is arguably much easier when only considering defaulters as compared to considering both timely-repaying and non-performing costumers. Digital lenders appear to have few options to stimulate repayment other than using dynamic incentives, as digital credit neither requires collateral nor is it economically worthwhile to file law suits for defaults on the small-sized digital credit. Even dynamic incentives might only decrease first time defaults but, perversely, increase the number of strategic defaults in the longer run [21]. Blacklisting hence constitutes a valuable additional enforcement mechanism. Furthermore, the cost of sharing information about non-performing costumers, arguably, is rather low. It is therefore likely that, in the view of many lenders, discouraging strategic default through strict blacklisting might outweigh the indirect cost of sharing information about risky borrowers. Indeed, our results show (Table 2) that the ratio of reported defaults is, on average, considerably higher among digital lenders (38.4%) compared to both formal (22.7%) and semi-formal lenders (2.1%). It should be noted that the small sample size of defaults on formal credit causes the respective standard error to be quite large. We still view this as an indication that the lender´s behavior has considerable influence on high blacklisting rates in the digital credit market, even if one assumed that default rates were entirely caused by borrowers’ behavior.

## 6. Policy recommendation and conclusion

Many individuals in emerging and developing economies are still credit rationed from conventional (semi-) formal credit markets, which is considered an extensive impediment to the development of these economies. Digital credit, as computer-aided mobile- and information technology, has sparked new enthusiasm to overcome problems of conventional lenders in reaching large portions of individuals in developing economies, thereby increasing credit access and consequently contributing to the economic development [10, 11].

In this article, we showed that digital credit did indeed reach a substantial share of Kenya’s population. Many of those who take up digital credit have characteristics that make them less likely to have access to the conventional formal credit market. However, we also show that digital credit, up until now, has not shown to be a substantive pathway to conventional formal credit nor does it appear sustainable in its current form, due to considerably higher default and blacklisting rates compared to conventional semi-formal and formal credit. The latter finding suggests that digital borrowing could even lead to insurmountable hurdles to full credit access rather than constitute a gateway to it.

These results offer important insights for policy makers and researchers. In the following, we formulate regulations and measures that should help the generally promising innovation of digital credit to increase credit access for millions of un- or underserved individuals in developing countries in a potentially more sustainable manner.

### 6.1 Default reducing measures

While default in the digital credit market considerably contributes to high blacklisting rates and thereby threatens the sustainability of that market, this article finds some indication that a mere self-selection of risky borrowers into the digital credit market seems insufficient in explaining differences in default compared to the other credit markets. In line with that finding, grey literature implies that high default rates in the digital credit market may be caused by both the lender’s and/or the borrower’s behavior with the relative contribution respectively being unknown. Thus, we strongly recommend more research to be conducted in order to understand why default rates are considerably higher in the digital credit market, thereby giving way to design tailor-made measures to decrease default rates, a necessary condition for a sustainable digital credit market.

### 6.2 An all-encompassing reporting

Further, we not only find that blacklisting rates among defaulters are especially high in the digital credit market but also that digital credit, up until now, does not constitute a gateway to formal credit. The findings indicate that digital lenders do not fully report both positive and negative repayment behavior, despite existing regulations requiring lenders to do so. Our findings are therefore in line with previous grey literature [5, 39], pointing out that existing regulations may not be fully enforced. An enforced all-encompassing reporting would give way to credit scores, which are not subject to strategic considerations of lenders about what information to share but express an objective reporting of a person’s overall repayment behavior. Such credit scores should then constitute a part in a lender´s decision of which credit terms and conditions to offer to an applicant. To enforce all-encompassing reporting and thereby increase consumer protection, lenders could be required to inform digital borrowers, e.g. via SMS, about all information shared, which, contrasting to the status quo, would not only encompass informing about negative but also about positive reports. Enforcing all-encompassing reporting could also have a positive effect for lenders since borrowers may be more willing to repay a credit when they are sure that a positive report will be sent to a credit bureau. Further, it is important to ensure that digital borrowers have easy and free access to the overall borrowing history as reported to the CRBs, so that the borrowers can control the lenders´ claims about what has been reported.

If all-encompassing reporting is enforced, blacklisting becomes a mere manifestation of default, i.e. a negative report in the overall borrowing history. A risk of enforcing to report every default is that lenders continue to use defaults as heuristics to assign credit worthiness rather than looking at the overall borrowing history, with the consequence that the overall number of blacklisted individuals simply rises given that default ratios maintain constant. Momentarily, borrowers in Kenya receive either a credit status or a credit clearance certificate, where the former reveals that the borrower has had a negative report in the past and the latter is only accessible to non-defaulters. Such a differentiation into two different certificates, might cause lenders to continue making their lending-decisions based on a binary measure (i.e. defaulter vs. non-defaulter) rather than based on a more sophisticated credit score.

While we see the contributions of this paper as important, it is also exploratory and descriptive in nature and consequently not without limitations. First, the present study uses self-reported data on borrowing behavior, default and blacklistings. It is plausible that default rates and blacklistings may be systematically underreported since the failure to repay a credit is socially frowned upon [8]. Future research should thus also compare our findings to data on default and blacklisting rates from credit providers and official institutions, which collect data on the aforementioned measures. However, as individuals are expected to underreport their actual default rates, we would only expect the here-described threats to the sustainability of digital credit to be even larger and therefore any potential bias arising from self-reporting is unlikely to reverse or negate the core results of the present study.

Second, this article uses nationally representative data for Kenya. Even though Kenya is a paradigm for digital credit usage, socio-cultural, political and other relevant differences should be considered when results are transferred to other countries. We therefore strongly encourage future research to investigate default and blacklisting rates of digital credit and their underlying mechanisms in other contexts as well.

Third, the present study cannot make any causal claims on the precise reasons as to why default rates are considerably higher in the digital credit market compared to conventional credit markets. Understanding the underlying mechanisms, especially to which extend credit providers or borrowers cause the high default rates should be paramount for future research. Similarly, even though the present study finds a strong indication that digital credit providers not only have an incentive to underreport positive borrowing behavior and compared to other lenders more likely report negative borrowing-behavior but also act accordingly, the data used in this study does not allow us to make causal claims about the role of the lender’s behavior and leaves that investigation to future research.
